# In Vitro Antimicrobial and Anticancer Peculiarities of Ytterbium and Cerium Co-Doped Zinc Oxide Nanoparticles

**DOI:** 10.3390/biology11121836

**Published:** 2022-12-16

**Authors:** Essia Hannachi, Firdos Alam Khan, Yassine Slimani, Suriya Rehman, Zayneb Trabelsi, Sultan Akhtar, Ebtesam A. Al-Suhaimi

**Affiliations:** 1 Department of Nuclear Medicine Research, Institute for Research and Medical Consultations (IRMC), Imam Abdulrahman Bin Faisal University, P.O. Box 1982, Dammam 31441, Saudi Arabia; 2 Department of Stem Cell Research, Institute for Research and Medical Consultations (IRMC), Imam Abdulrahman Bin Faisal University, P.O. Box 1982, Dammam 31441, Saudi Arabia; 3 Department of Biophysics, Institute for Research and Medical Consultations (IRMC), Imam Abdulrahman Bin Faisal University, P.O. Box 1982, Dammam 31441, Saudi Arabia; 4 Department of Epidemic Diseases Research, Institute for Research and Medical Consultations (IRMC), Imam Abdulrahman Bin Faisal University, P.O. Box 1982, Dammam 31441, Saudi Arabia; 5 Department of Physics, Faculty of Science of Bizerte, University of Carthage, Bizerte 7021, Tunisia; 6 Biology Department & Institute for Research and Medical Consultations (IRMC), College of Science, Imam Abdulrahman Bin Faisal University, P.O. Box 1982, Dammam 31441, Saudi Arabia

**Keywords:** ZnO nanoparticles, rare earth, doping, antimicrobial activity, anticancer activity

## Abstract

**Simple Summary:**

Nanotechnology is an emerging interdisciplinary research field that brings together materials science, engineering, chemistry, biology, and medicine. There is no doubt that nanomedicine has an amazing potential for early detection, optimal diagnosis, and personalized cancer treatment. In recent years, nanoparticles have been widely used in biomedical applications, and among the metal oxides nanomaterials, zinc oxide (ZnO) nanoparticles have shown unique physical and chemical properties. Furthermore, ZnO is less toxic and cheaper, making it a suitable candidate for drug delivery, bioimaging, wound healing, antimicrobial applications, and cancer treatment. The ZnO-doped nanomaterials showed unprecedented properties with respect to their pure material counterparts. In the present work, we propose to study the anticancer and antimicrobial activities of ZnO doped with the rare earth elements Yb and Ce. We found that samples doped with x = 0.01 and x = 0.05 of Yb and Ce showed a better inhibitory effect on HCT-116 cancer cells than unadded ZnO (x = 0.00). In addition, the treatment of nanoparticles doped with Ce and Yb induced apoptosis in HCT-116 cells. In summary, our results demonstrated that the synthesized nanoparticles showed antifungal, antibacterial and anticancer potential, which could be considered for potential pharmaceutical applications.

**Abstract:**

Zinc oxide nanoparticles (ZnO NPs) are a promising platform for their use in biomedical research, especially given their anticancer and antimicrobial activities. This work presents the synthesis of ZnO NPs doped with different amounts of rare-earth ions of ytterbium (Yb) and cerium (Ce) and the assessment of their anticancer and antimicrobial activities. The structural investigations indicated a hexagonal wurtzite structure for all prepared NPs. The particle size was reduced by raising the amount of Ce and Yb in ZnO. The anticancer capabilities of the samples were examined by the cell viability MTT assay. Post 48-h treatment showed a reduction in the cancer cell viability, which was x = 0.00 (68%), x = 0.01 (58.70%), x = 0.03 (80.94%) and x = 0.05 (64.91%), respectively. We found that samples doped with x = 0.01 and x = 0.05 of Yb and Ce showed a better inhibitory effect on HCT-116 cancer cells than unadded ZnO (x = 0.00). The IC_50_ for HCT-116 cells of Ce and Yb co-doped ZnO nanoparticles was calculated and the IC_50_ values were x = 0.01 (3.50 µg/mL), x = 0.05 (8.25 µg/mL), x = 0.00 (11.75 µg/mL), and x = 0.03 (21.50 µg/mL). The treatment-doped ZnO NPs caused apoptotic cell death in the HCT-116 cells. The nanoparticles showed inhibitory action on both *C. albicans* and *E. coli*. It can be concluded that doping ZnO NPs with Yb and Ce improves their apoptotic effects on cancer and microbial cells.

## 1. Introduction

Nanoscale materials are widely applied in various research fields—especially biology, physics, and chemistry—due to their specific and novel properties that do not appear in the bulk phase [1,2,3]. Zinc oxide (ZnO), titanium oxide (TiO_2_), and iron oxide (Fe_2_O_3_) are the most frequently-utilized metal oxides (MOx) in biomedical applications. Ideally, the MOx NPs should fulfill the following features: (i) chemically stable, (ii) biocompatible, (iii) resistant to scratch and wear, and (iv) nontoxic. Inorganic zinc oxide (ZnO) nanomaterial is known for its low cost [4]. A preliminary economic evaluation study for the production of a large amount of ZnO NPs using the sol-gel route has been recently reported. Compared to different economic factors, the study turned out to be beneficial and profitable by raising the cost of the raw elements by less than 100% of their cost and revealing the result of potential fabrication to meet the demand of ZnO NPs [5]. In addition, ZnO is characterized by its biocompatibility and bioactivity [4]. From a biological and biomedical point of view, ZnO is categorized as a “GRAS” (generally recognized as safe) material by the Food and Drug Administration (FDA) [6]. ZnO is also a suitable alternative to titanium dioxide (TiO_2_) [7,8]. For instance, G.Y. Nigussie et al. performed a comparative study on the antibacterial activity of ZnO and TiO_2_ doped with Ag. Their results showed that at a concentration of culture of 60 μg/mL, both TiO_2_- and ZnO-doped NPs were toxic to *E. coli*, *S. aureus*, and *P. aeruginosa* bacteria. However, the efficiency of ZnO-doped NPs is higher than those of TiO_2_-doped NPs [9].

The original properties of ZnO NPs are highly dependent on the particle size, composition, crystal structure, shape, and morphology. These properties are why their synthesis has aroused much attention and various synthesis processes have been developed to obtain high-quality NPs with the desired physical and chemical features [10,11]. Different synthesis methods have been proposed to produce ZnO and doped ZnO NPs such as the coprecipitation method [12], the hydrothermal method [13], the sol-gel method, the microwave-assisted method [14], and the solid-state reaction [15]. Among the different methods, the sol-gel route presents many advantages such as good stoichiometric control in precursor solutions, facility of compositional changes, customizable microstructure, relatively low annealing temperatures, and simple and cheap equipment [16].

During the last decades, the use of ZnO NPs has rapidly invaded areas such as optoelectronics [17], biomedicine [18], environmental protection [19], cosmetics [20], and photocatalysis [21], among others. ZnO is also recognized as an n-type semiconducting material that exhibits a large bandgap (~3.3 eV) and a high exciton binding energy (~60 meV) [22]. Moreover, ZnO displays peculiar physical and chemical properties such as photocatalytic activities [23,24], pyroelectric [25], and piezoelectricity behaviors [26]. These properties are useful in the medical field since, in its nanoscale size, ZnO shows other convincing properties that allow effective antimicrobial activity and strong toxicity against various kinds of pathogenic bacteria such as *Klebsiella pneumoniae*, *Shigella dysenteriae*, *Escherichia coli*, *Pseudomonas aeruginosa*, *Proteus vulgaris*, *Staphylococcus aureus*, and *Streptococcus pneumoniae* [27,28,29]. Due to these features along with the biosafety and chemical stability of ZnO NPs, they are well suited for the selective inhibition of cancer cells through the production of reactive oxygen species (ROS) [30].

With the emergence of new cancers as well as several virulent pathogenic species causing infections and severe diseases, improving the functionalities of the ZnO NPs is indispensable. Hence, further attempts are needed to render ZnO more appealing. One powerful method to achieve this purpose is chemical doping, in which the electrical [31], electromechanical [32], antibacterial [33], and optical [31] properties can be improved after doping ZnO with suitable transition metals elements such as iron (Fe), vanadium (V), cobalt (Co), copper (Cu), and chromium (Cr). Furthermore, adding impurities through the doping approach has been reported in several studies over the last decade. The results revealed that, after doping, ZnO NPs became more effective in their antimicrobial, antibacterial, and anticancer activities than pure ZnO. For example, Pd and Au noble metals have been tested as ZnO doping agents and have shown very promising results for their antibacterial activity against *Escherichia coli* (*E. coli*) [34]. In addition, a very recent study showed that Ag-doped ZnO constitutes a promising candidate for having anticancer and antibacterial activities due to the increased generation of ROS [35]. However, these metals are costly. Considering their economic value, rare earth (RE) elements such as La, Ce Eu, Gd, etc., are another appropriate choice for ZnO doping as they add interesting features to the material. Indeed, RE-doped ZnO NPs are already used to inhibit microbial growth, providing a significant reinforcement in anticancer and antibacterial efficacy [36,37,38,39]. It has also been confirmed that RE-doped ZnO NPs constitute very potent biomarkers for early cancer diagnosis [40,41]. Many previous works have investigated the impact of individual RE elements on the properties of ZnO [42,43,44]. It has been reported that the nature of dopants plays a leading role in the performance of ZnO. For instance, Yuanzhe Li et al. [45] prepared ZnO NPs doped with different rare-earth elements such as Pr, Gd, Ce, and La using the wet chemical method to study their antimicrobial activity. Their findings showed that La-doped ZnO exhibited the highest antibacterial activity with a rate of 85% against *E. coli* and Pseudomonas aeruginosa (*Pseudomonas*). In addition, Ce-doped ZnO NPs presented a good bactericidal rate of 83% after a 25-min UV exposure. M. Karthikeyan et al. [46] performed a comparative study on the individual doping effect of three types of RE elements including Nd, La, and Ce on the antibacterial and anticancer features of ZnO. A minimal toxicity percentage was obtained for all ZnO-doped samples using Vero cells.

Among the diverse doping opportunities, cerium—which belongs to the rare earth family—is an appealing element. The use of cerium can provide interesting characteristics for the doped nanomaterials such as ZnO because of the 4f orbital availability and ionic Ce–O bond bonding [47]. Cerium exhibits various oxidation states such as Ce^3+^/Ce^4+^, which can show different optical and electronic features and form oxygen vacancies [48]. The 4f orbital of the cerium atom interferes with the Zn 3d and O 2P orbital, which leads to a novel localized band that arises between the conduction and valence bands of ZnO by cerium doping. A bridge can therefore be formed between the conduction and valence bands. This could improve the optical performance of ZnO. In addition, cerium is attracting much interest as a suitable biomaterial due to its ability to cause oxidative stress in irradiated cancer cells without altering normal cells [49]. C. Theivarasu et al. studied the impact of Ce^3+^ ions on the anticancer and antibacterial activities of ZnO NPs prepared by the coprecipitation route [50]. ZN Kayani et al. demonstrated a good antibacterial activity of Ce-doped ZnO thin films [51]. On the other hand, the Yb ion displays the highest electron affinity [52]. When mixed with other RE elements such Eu^3+^ or Er^3+^, Yb^3+^ is employed as a sensitizer and hence improve the up-conversion (UC) luminescence productivity of ZnO [53]. A recent work showed that doping ZnO NPs with Yb can be an effective way to enhance its antimicrobial activity against *E. coli* and Staphylococcus aureus and minimize some common side effects [54].

To our knowledge, there are no reports regarding the combined effect of cerium and ytterbium rare earth elements on the bioactivities of ZnO NPs. In this paper, we report the influence of the combination of cerium (Ce) and ytterbium (Yb) elements as co-dopants on the anticancer and antimicrobial properties of ZnO nanoparticles. In this regard, we aim to synthesize ZnO NPs co-doped with Ce and Yb using the sol-gel autocombustion method. The structure, morphology, anticancer, and antimicrobial activities of these prepared co-doped NPs were investigated and discussed in detail.

## 2. Materials and Methods

### 2.1. Chemicals

The chemicals used were zinc nitrate hexahydrate [Zn(NO_3_)_2_·6(H_2_O); 99% (used as the source of zinc and provided from Techno Pharmchem, New Delhi, India)], cerium (III) nitrate hexahydrate [Ce(NO_3_)_3_·6(H_2_O); 99% (provided from Loba Chemie, Mumbai, India)], and ytterbium (III) nitrate pentahydrate [Yb(NO_3_)_3_·5(H_2_O); 99.9% (provided from Sigma Aldrich, St. Louis, MI, USA)] as the starting materials. All reagents were used without further purification. Citric acid (99%) was used as a fuel, and deionized water (DI) was used as the solvent.

### 2.2. Synthesis

The sol-gel autocombustion route was adopted for the fabrication of pure (x = 0.0) and (Yb, Ce) co-doped (x = 0.01, 0.03, and 0.05) ZnO nanoparticles. Initially, 6g of Zn(NO_3_)_2_·6(H_2_O) was dissolved in 30 mL of DI water. Then, proper amounts of Ce(NO_3_)_3_·6(H_2_O) and Yb(NO_3_)_3_·5(H_2_O) were added to the above solution according to the doping concentration, which was followed by constant stirring at 80 °C on the hot plate of a magnetic stirrer for 1 h to obtain a homogenous solution. Meanwhile, citric acid was prepared in another beaker and then added to the prepared solution with continuous stirring. The pH of the solution was adjusted at 7. The solution was stirred on a hot plate and the gradual evaporation of water occurred until a gel appeared, which turned into a foam that eventually burned spontaneously to give rise to ashes. The obtained ashes were collected, ground, and then placed into the furnace to be calcined at 500 °C for 4 h to remove volatile substances or remaining organic groups. The calcination step was completed under air atmosphere with a heating rate of 3 °C/min followed by furnace cooling to form bare ZnO and Yb and Ce co-doped ZnO NPs.

### 2.3. Characterization

To study the crystal structure of the fabricated specimens, the XRD technique using a Rigaku MiniFlex X-ray diffractometer with Cu Kα radiation was used. The data were registered in the 20–80° 2θ range and refined by the Rietveld method by using Match 3! software. The surface morphology and chemical atomic percentages were reached via a Tescan Vega scanning electron microscope with an EDX detector. The microstructure and particle size distribution were examined using a Morgagni 268 FEI transmission electron microscope (TEM, Czech Republic).

### 2.4. Antimicrobial Assay

The fungal strain *C. Albicans* ATCC 14,053 and the bacterial strain *E. coli* ATCC35218 (Gram-negative bacteria) were selected to study the antimicrobial action of different samples. Using a shaking incubator, *C. Albicans* and *E. coli* were freshly grown in Sabaurauds and nutrient broth (MOLEQULE-ON, cat no: MM-M-060-500) overnight at 28 and 37 °C, respectively. Cell pellets were washed with phosphate-buffered saline several times. Then, they were diluted with NaCl solution (0.9%) to achieve cell concentrations of 107 CFU/mL for further studies. The standard broth dilution method was used to examine the antimicrobial action of Yb and Ce co-doped ZnO NPs. MIC was studied by culturing the fungi and bacteria in SDB and Mueller Hinton broth (Scharlau 02-136-500), respectively, with the prepared nanoparticles with a concentration ranging from 0.25 mg/mL to 4 mg/mL. The control (without the nanoparticles) was also involved in this work. The MIC represents the lowest test drug concentration that inhibits the organism’s growth. The aliquots of the treated fungi and bacteria were placed on the freshly-ready SDA and MHA dishes for overnight incubation at 37 °C. The minimum concentration of nanoparticles in which there is no growth or CFUs lower than three cells was considered as the MFC/MBC.

### 2.5. Anticancer Activity

Cancer cells (human colorectal carcinoma HCT-116) and normal cells (human embryonic kidney cells HEK-293) were purchased from American Type Cell Culture (ATCC), USA and were considered to study the action of Ce and Yb co-doped nanoparticles on cell viability and cell proliferation. As per the previously-described method [31], the cells were grown in a medium containing DMEM (Gibco, Waltham, MA, USA), NEAA (Invitrogen, Waltham, MA, USA), fetal bovine serum (Invitrogen, Waltham, MA, USA), L-glutamine (Invitrogen, Waltham, MA, USA), penicillin (Invitrogen, Waltham, MA, USA), streptomycin (Invitrogen, Waltham, MA, USA), and selenium chloride (Invitrogen, Waltham, MA, USA). These samples were kept in the 5% CO_2_ incubator and when cells become confluent, they were treated for an MTT assay.

#### 2.5.1. MTT Assay

A total of 300,000 cells/mL were seeded for the MTT assay. The cells were treated with all prepared nanoparticles (2.0 µg, 10 µg, 20 µg and 30 µg/mL) for 48 h and kept in the CO_2_ incubator. In the control wells, the prepared nanoparticles are not included. The control, ZnO (x = 0.00), and doped ZnO samples were treated with 5.0 mg/mL of MTT. The cells were kept in the incubator of CO_2_ for 4 h. Finally, cells were washed with culture medium, then DMSO (Sigma Chemicals, Schnelldorf, Germany) was added and the culture plate was assessed using a Bio-Tek plate reader (USA) at a wavelength of 570 nm. We then calculated the percentage of cell viability for the statistical analysis as conducted by Dennett’s post hoc test with GraphPad Prism Software version 6.0 USA. The percentage of cell viability was calculated using the following formula: cell viability (%) = OD sample/OD control × 100. The data were analyzed using one-way ANOVA and Tukey’s test. All statistics were conducted using GraphPad Prism Software version 6.0 USA. A *p*-value less than 0.05 was considered to be statistically significant.

#### 2.5.2. Cancer Cell Nuclear Staining

To study the impact of the prepared nanoparticles on the cancer cell nucleus for the apoptotic cell death, cancer (HCT-116) cells were analyzed by DAPI staining. In the control group, the prepared nanoparticles were not included, while, in the experimental group, ZnO (x = 0.00), and co-doped ZnO NPs (30 µg/mL) were included. After treatment for 48 h, the cells were treated with 4% paraformaldehyde as a fixative, washed in PBS, and finally stained in DAPI (1.0 μg/mL) to be cover-slipped. The blue color fluorescent DAPI was observed using Confocal Scanning Microscope (Zeiss, Jena, Germany).

## 3. Results

### 3.1. SEM and TEM Analysis

**Figure 1:**
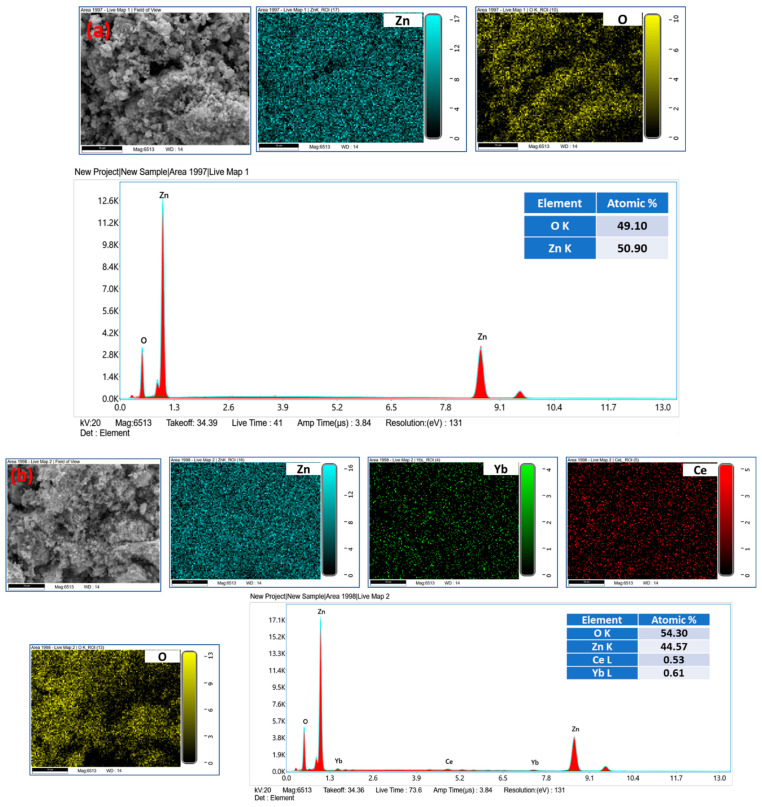

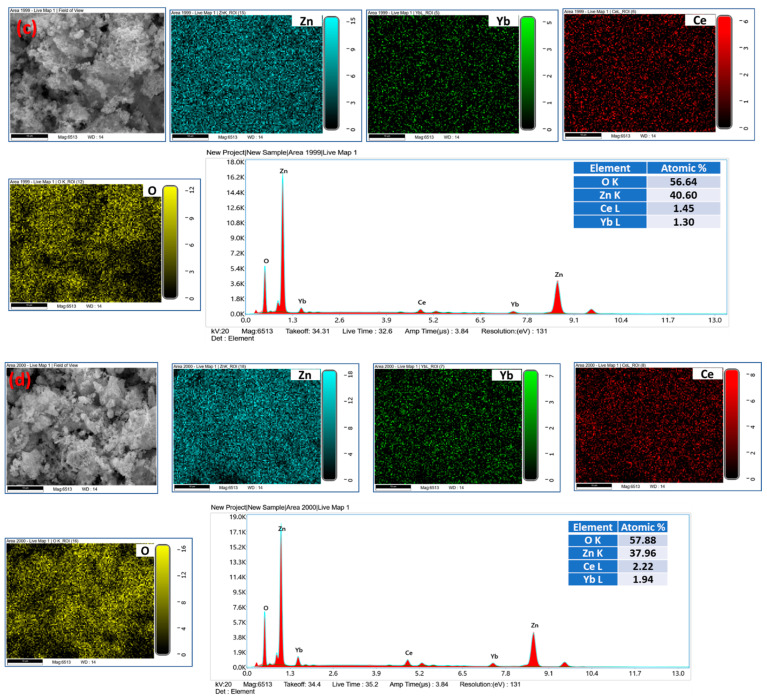
SEM images, elemental mapping, EDX spectra, and chemical compositions of (**a**) 0.0, (**b**) 0.01, (**c**) 0.03, and (**d**) 0.05 Yb and Ce co-doped ZnO NPs.

**Figure 2:**
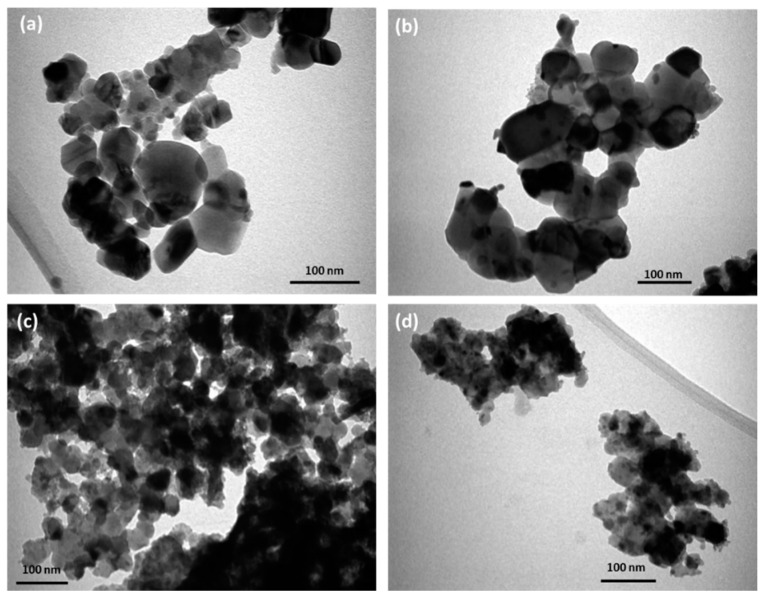
TEM images of (**a**) 0.0, (**b**) 0.01, (**c**) 0.03, and (**d**) 0.05 for Yb and Ce co-doped ZnO NPs.

Figure 1a–d show the surface morphologies and the elemental mapping of different samples. The SEM morphologies were presented under the same magnification. The morphology of pure ZnO consists of irregular nanoparticles. The morphology evolution of ZnO due to Ce and Yb dopants maintains the non-uniformity of the prepared nanopowders. To inspect the elemental composition, the samples were assessed by energy dispersive spectroscopy (EDS) attached with SEM. The typical EDS spectrum as well as the elemental compositions of the pure ZnO sample (Figure 2a) shows that the sample is composed of two major elements, namely Zn, and O, while that of doped Yb and Ce ZnO (Figure 2b–d) confirms the presence of Ce and Yb in addition to the above-mentioned elements (Zn, O).

**Figure 3:**
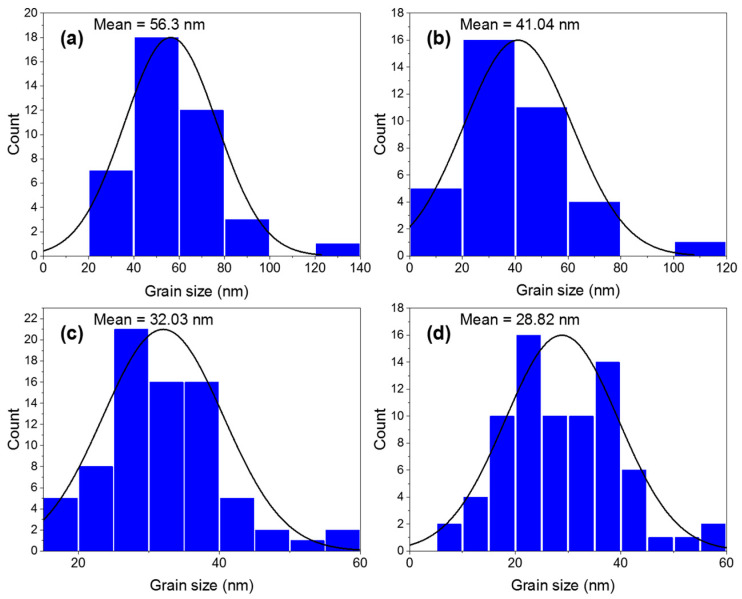
Histograms of (**a**) 0.0, (**b**) 0.01, (**c**) 0.03, and (**d**) 0.05 for Yb and Ce co-doped ZnO NPs.

In addition, TEM observations were performed. TEM images and particle size distributions of pure and doped ZnO nanoparticles are shown in Figure 2 and Figure 3. The images of x = 0.00 and x = 0.01 doped samples show a nearly hexagonal shape with a mean particle size of 56.3 and 41.4 nm, respectively. The average particle sizes decrease from 56.3 nm for 0.00 to 41.4, 32.03, and 28.82 nm for x = 0.01, x = 0.03, and x = 0.05, respectively.

### 3.2. XRD Study

**Figure 4:**
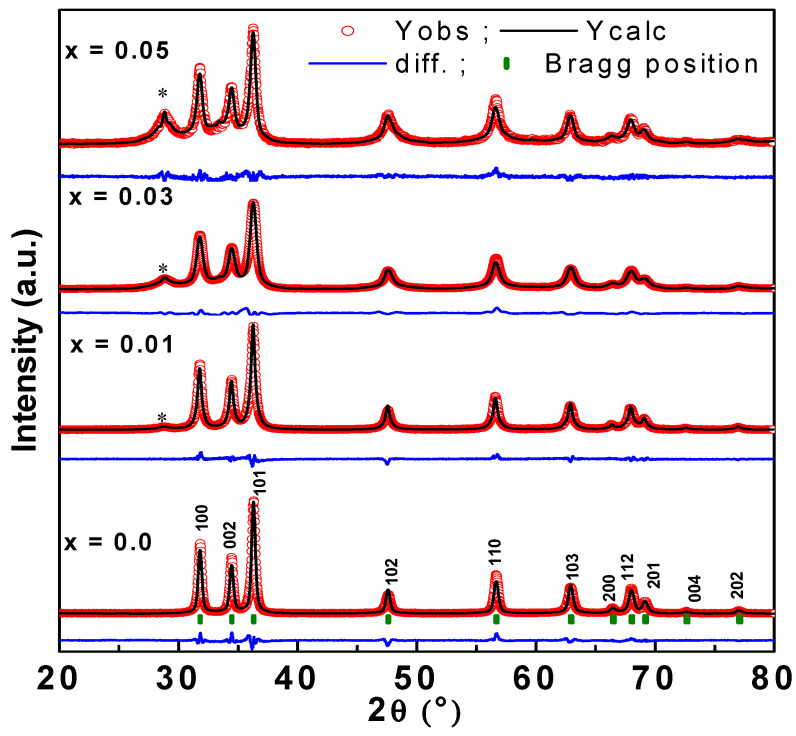
XRD patterns of Yb and Ce co-doped ZnO NPs where ‘*’ indicates the peaks belonging to a secondary phase.

The structural properties of the different prepared samples have been investigated in depth elsewhere [55] and here, we present an overview of the phase formation and its evolution with doping effects. All the samples have nine diffraction peaks positioned at 31.78°, 34.44°, 36.24°, 47.62°, 56.64°, 62.92°, 66.48°, 68.00°, 69.22°, 72.60°, and 76.98° associated with the (100), (002), (101), (102), (110), (103), (200), (112), (201), (004), and (202) planes, respectively (Figure 4). The obtained XRD peaks for all samples were assigned to the most thermodynamically ZnO stable phase—the wurtzite hexagonal structure—with the corresponding space group of P63mc. For co-doped samples, a minor second phase, which matches with the secondary phase of Yb_0.3_Ce_0.7_O_1.85_, was detected.

The lattice constants (a and c) of different as-prepared specimens were calculated by least-square Rietveld refinement analysis [56] and their values are listed in Table 1. The crystallite size (*D*) was calculated using the Scherrer’s equation $\left(D=\frac{k\lambda }{\beta cos\theta }\right)\;$ [56]. *β* denotes the full width at half maximum FWHM in radian, *k* is a dimensionless constant (0.9), *λ* is the X-ray wavelength, and *θ* is the Bragg angle in degree. The crystallite sizes of pure ZnO (0.00), ZnO: Yb-Ce (0.01), ZnO: Yb-Ce (0.03), and ZnO: Yb-Ce (0.05) samples were found to be 23.76, 21.33 nm, 19.34 nm, and 18.09 nm, respectively.

### 3.3. Antimicrobial Action

**Figure 5:**
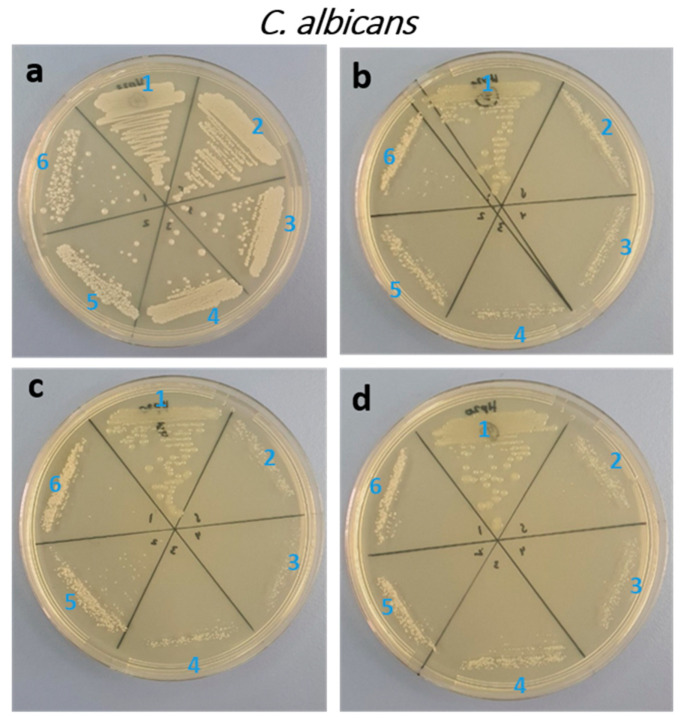
Agar plates showing the MIC/MFC of *C. albicans* upon treatment with Yb and Ce co-doped ZnO NPs (x = 0.00, x = 0.01, x = 0.03, x = 0.05). ((**a**): x = 0.00, (**b**): x = 0.01, (**c**): x = 0.03, (**d**): x = 0.05), (1: untreated; 2: 0.25; 3: 0.5; 4: 1; 5: 2; 6: 4 mg/mL).

In the current study, the antifungal and antibacterial actions of Ce and Yb co-doped ZnO nanoparticles against *C. albicans* and *E. coli* were studied by assessing MIC and MFC/MBC, respectively. The MBC and MIC of pure ZnO and doped ZnO nanoparticles against the *C. albicans* are summarized in Figure 5 and Table 2.

**Figure 6:**
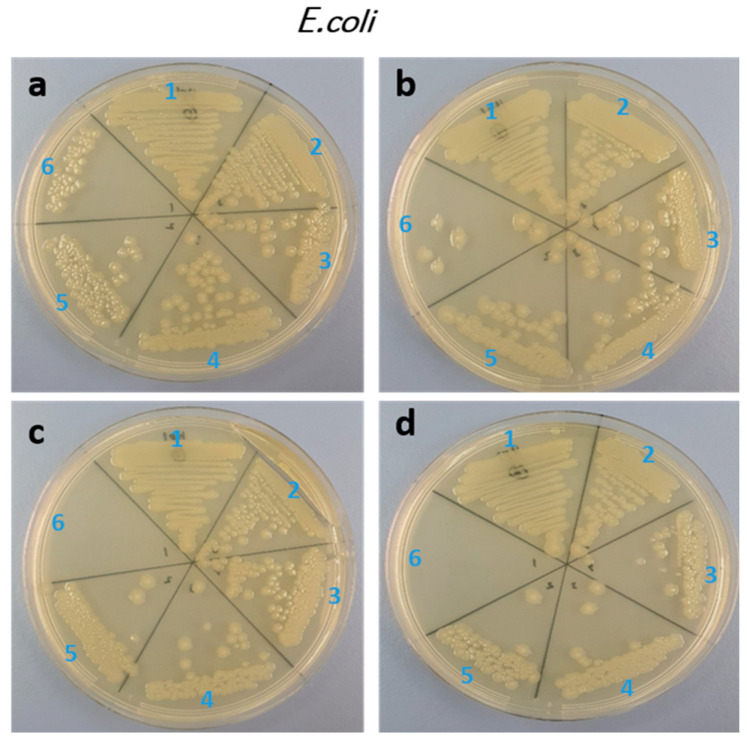
Agar plates showing the MIC/MBC of E.Coli upon treatment with Yb and Ce co-doped ZnO NPs (x = 0.00, x = 0.01, x = 0.03, x = 0.05), ((**a**): x = 0.00, (**b**): x = 0.01, (**c**): x = 0.03, (**d**): x = 0.05), (1: untreated; 2: 0.25; 3: 0.5; 4: 1; 5: 2; 6: 4 mg/mL).

The minimum MIC values were registered as 1, 0.25, 0.25, and 0.25 mg/mL for x = 0.00, x = 0.01, x = 0.03, x = 0.05, respectively. However, the MFC was higher than 4 mg/mL in all samples. The antibacterial activity was seen moderately higher against *E. coli*. The MIC was 2, 1, 1, 0.5 mg/mL for x = 0.00, 0.01, 0.03, 0.05, respectively. However, the MBC of 4 mg/mL was recorded with all the ratios (Table 2) and (Figure 6).

### 3.4. Cancer Cell Viability

**Figure 7:**
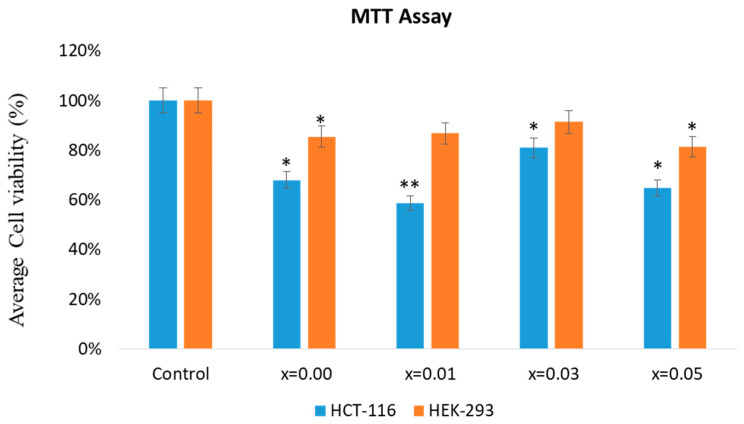
Cell viability using the MTT assay. The impact of the treatment of Yb and Ce co-doped ZnO on the colon cancer (HCT-116) cells and normal kidney (HEK-293) cells stained post 48-h treatment. * *p* < 0.05; ** *p* < 0.01.

The impact of pure ZnO (x = 0.00), and doped ZnO on HEK-293 and HCT-116 cells was evaluated. As depicted in Figure 7, a noteworthy reduction in the cell viability is seen after treatment with Ce and Yb co-doping. The inhibitory concentration (IC_50_) of Ce and Yb co-doped ZnO NPs was also calculated. The anticancer capabilities of the prepared nanoparticles were assessed by a cell viability MTT assay. Post 48-h treatment showed a considerable reduction in the cell viability after the NPs treatment. The cell viability was 68%, 58.70%, 80.94% and 64.91% for ZnO (x = 0.00), ZnO: Yb-Ce (0.01), ZnO: Yb-Ce (0.03), and ZnO: Yb-Ce (0.05), respectively. We found that ZnO co-doped with x = 0.01 and x = 0.05 of Yb and Ce displayed better inhibitory actions on the cancer cells than ZnO (x = 0.00). The IC_50_ for HCT-116 cells of Ce and Yb co-doped ZnO nanoparticles was calculated and the IC_50_ values were x = 0.01 (3.50 µg/mL), x = 0.05 (8.25 µg/mL), x = 0.00 (11.75 µg/mL) and x = 0.03 (21.50 µg/mL), respectively. The impact of the prepared nanoparticles on HEK-293 normal cells was evaluated, and the cell viability was 86%, which was superior to HCT-116 colon cancer cells, for which the viability was 68% (Figure 7).

**Figure 8:**
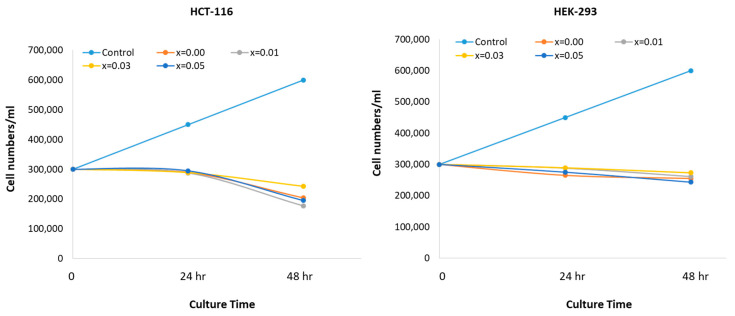
Cell growth curve shows the impact of treatment of Yb and Ce co-doped ZnO on the colon cancer (HCT-116) cells and normal kidney cells (HEK-293) cells post-48-h treatment.

ZnO co-doped with x = 0.01, and x = 0.05 of Yb and Ce displayed better inhibitory actions on the cancer cells than ZnO (x = 0.00). This effect may be attributed to the difference in the average particle size of nanoparticles as revealed by the TEM analysis, which showed that the average particle sizes of x = 0.01 and x = 0.05 were smaller than Xs nanoparticles. There are few reports which suggested that the size of the nanoparticles plays a critical role in cancer cell penetration, with smaller sizes showing better cancer cell penetration [57,58]. Cell growth curves after the treatment of Yb and Ce co-doped ZnO on the colon cancer (HCT-116) cells and normal kidney cells (HEK-293) are depicted in Figure 8.

### 3.5. Cancer Cell DNA Disintegration

**Figure 9:**
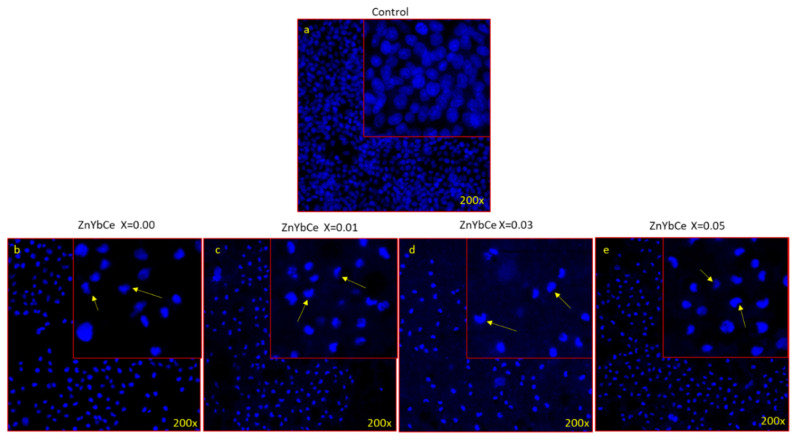
(**a**–**e**) Cancer cells morphology by DAPI staining. The impact of treatment with Yb and Ce co-doped ZnO NPs (x = 0.00, x = 0.01, x = 0.03, x = 0.05) on HCT-116 cells stained with DAPI post 48-h treatment. (**a**) (control) cell nucleus remains intact and normal, (**b**–**e**) are treated with nanoparticles. Images were taken by a fluorescence confocal microscope. The cells treated with nanoparticles showed chromatin condensation (arrows) which is the feature of apoptotic cell death.

To identify the nuclear morphology due to apoptosis, DAPI staining was adopted. The blue color DAPI staining stains particularly nuclei dye [59]. The staining also permits the transformations in nuclear structure including chromatin fragmentation and chromatin condensation owing to apoptosis induced by toxic or nanoparticle treatment. In the current work, we found that cancer cells treated without Ce and Yb co-doped nanoparticles displayed round and normal nuclei. No indication of fragmentations and chromatin condensation was seen in this set (Figure 9a). However, the nuclei of cancer cells treated with Ce and Yb co-doped nanoparticles exhibited chromatin condensation and fragmentation (Figure 9b–e).

## 4. Discussion

In the current work, the combined effect of Ytterbium and cerium doping on the structural, morphological, antibacterial and anticancer properties were studied. It is evident that Ce and Yb co-doping affected the structural parameters of ZnO NPs. An increase in the unit cell volume (V) was obtained for Yb and Ce co-doped samples compared to nondoped ZnO sample. This result is mostly ascribed to the mismatch in the ionic radii of the Yb (0.86 Å), Ce^3+^/Ce^4+^ (1.01 Å/0.92Å), and Zn (0.74 Å) elements [60]. Indeed, the ionic radii of Yb and Ce ions is higher than that of Zn cations, and the unit cell volume expansion of ZnO indicates that some of the Yb and Ce ions might be included in the interstitial sites. Similar results were previously observed by M.A.M. Ahmed et al. [61]. The crystallite size decreased as the content of Yb and Ce increased. This effect can be associated with the mismatch in the ionic radii and the RE–O–Zn bond formation on the surfaces of NPs, which decrease crystallite growth [62]. Similar conclusions were reported by Diego E. Navarro-López et al. in the case of Zn_0.99_(Ce_0.01−x_M_x_)O (where M=Nd, Sm, and Er) nanoparticles [29]. The obtained results from the XRD are in good agreement with the TEM results. We observed an evident agglomeration of very small particles, especially in highly Yb-and Ce-doped samples. This result can be correlated to the formation of a minor phase (ytterbium cerium oxide in our case) and to the notable decrease in both the crystallite size and particle size as Yb and Ce dopants increased. E. Cerrato et al. reported the individual doping effects of different rare earth ions such as La, Ce, Pr, Er, and Yb on ZnO. The incorporation of RE ions in the ZnO structure caused an overall reduction in the crystallite size. The authors showed that the ZnO sample doped with Yb was the most affected, while the ZnO sample doped with Ce was the least affected. The authors attributed this effect to the formation of a cerium oxide phase in the case of Ce-doped ZnO. Their analysis using the TEM technique showed the presence of tiny particles anchored at the surface of ZnO in Ce-doped and Yb-doped ZnO samples, which was associated with cerium oxide in the case of Ce-doped samples and the sharpest reduction in crystallite size in the case of Yb-doped ZnO samples, respectively [63]. We observed the antibacterial and antifungal actions of Ce and Yb co-doped ZnO nanoparticles against *C. albicans* and *E. coli* by MIC and MFC/MBC, respectively. There are few studies on the treatment of cerium oxide, especially in nanoparticle form. Nanocerium has been reported to possess antibacterial properties mediated via oxidative stress [28]. In another study, Ce-doped ZnO nanoparticles showed inhibitory actions against *Staphylococcus aureus*, *Streptococcus pneumonia*, *Klebsiella pneumoniae*, *Shigella dysenteriae*, *Escherichia coli*, *Pseudomonas aeruginosa*, and *Proteus vulgaris* bacterial strains [38].

Due to the immense application of ZnO NPs, their stability is important for their biological activities. Therefore, measurements were carried out to obtain information about the stability of the prepared samples zeta potential. The zeta potential varied between 20.1 mV and 12.6 mV by Ce and Yb co-doping [55], which signified that the ZnO nanoparticles are stable. The decrease in the values of the zeta potential will lead to flocculation and aggregation due to van der Waals interparticle attraction. S. Anitha et al. [12] showed that the zeta potential of ZnO doped with Cu was 23.8–18.9 mV. A magnitude of 17.6 mV of zeta potential was obtained by S. Kavitha for ZnO NPs prepared by the co-precipitation method [64]. Hence, surface modifications are one of the techniques to enhance the interaction of ZnO NPs with biomolecules. Several strategies have been used to modify the ZnO NPs surface for the stability and activity that leads to enhanced dispersion and minimum aggregation [65,66,67]. The present study reports the modification using cerium and ytterbium that exhibits the enhancement in the action against the bacterium as well as Candida, which agrees with previous studies. Studies have previously reported the use of dopant ions in ZnO NPs to observe their relationships with the antibacterial and antifungal activity [68]. These dopant ions can reduce the size of ZnO NPs, which is an important characteristic for their antimicrobial action [69]. The mechanism of toxicity such as oxygen radicals, Zn^2+^ ions, and the interaction of nanocomposites with the cell surface, are greatly influenced by surface coatings that ultimately help in the interactions between nanocomposites and the cellular surfaces of bacteria and fungi [70,71]. A study by Ravichandran et al. reported enhanced antibacterial activity with ZnO:Cu:graphene. This was attributed to the smaller crystallite size, as with the smaller size, the number of ZnO NPs increased, and the increase in surface area that led to increased H_2_O_2_ generation [72].

This is the first report demonstrating an inhibitory action of synthesized nanoparticles co-doped with ytterbium and cerium against HCT-116 cells. The impact of prepared NPs showed a considerable reduction in the HCT-116 cell viability after the treatment. The cell viability was 68%, 58.70%, 80.94% and 64.91% for ZnO (x = 0.00), ZnO: Yb-Ce (0.01), ZnO: Yb-Ce (0.03), and ZnO: Yb-Ce (0.05), respectively. Ce- and Yb-doped ZnO nanoparticles with x = 0.01 and x = 0.05 showed the best results on cancer cells (HCT-116 cells). This may have been because these (x = 0.01 and x = 0.05) showed the lowest viability of cancer cells among other samples, which indicated the most inhibiting effect on cancer cells. The other samples showed inhibition, but the results were nonsignificant. Previous studies demonstrated that treatment of ZnO NPs produced inhibitory effects on cancer cells. In contrast, all powders of HAp:RE+ displayed a paramagnetic effect. Cell viability tests of several doped compounds were carried out such as HAp:Gd/Eu and HAp:Gd/Yb/Tm, and the stem cell culture of dental pulp showed their valid biocompatibility [73]. To identify the nuclear morphology due to apoptosis, DAPI staining was adopted. The blue color DAPI staining stains particularly nuclei dye [73]. The staining also permits the transformations in nuclear structure such as chromatin fragmentation and chromatin condensation owing to apoptosis induced by toxic or nanoparticle treatment. In the current work, we found that cancer cells treated without Ce and Yb co-doped nanoparticles displayed round and normal nuclei, and no indication of fragmentation and chromatin condensation was seen in this set. However, the nuclei of cancer cells treated with Ce and Yb co-doped nanoparticles exhibited chromatin condensation and fragmentation, indicating that our prepared nanoparticles showed programmed cell death (or apoptosis). There is a report where doped SnO_2_-ZnO/rGO NCs showed better anticancer activity on MCF-7 cells than nondoped ZnO nanoparticles [41]. The IC_50_ for ZnO NPs, SnO_2_-ZnO NPs, and SnO_2_-ZnO/rGO NCs were 54.61, 38.73, and 27.86 µg/mL for MCF-7 cells [34], whereas in the current investigation, the IC_50_ for HCT-116 of Ce and Yb co-doped nanoparticles was 3.50 µg/mL to 21.50 µg/mL, suggesting better inhibitory action on the HCT-116 cells with lower concentrations of Yb and Ce co-doping. Moreover, the SnO_2_-ZnO/rGO NCs induced an apoptotic response in MCF-7 cells through the upregulation of the caspase-3 gene and depletion of the mitochondrial membrane potential [34]. As a cytotoxic agent, ZnO NPs were more efficient at killing multidrug resistant (MDR) cancer cells, and the combination of ZnO NPs along with Doxorubicin showed high cytotoxicity in the 3D cancer cell spheroids. More importantly, it was demonstrated that ZnO NPs effectively down regulated CD44, a key cancer stem cell marker leading to successful killing of the cancer cells [74]. In another study, ZnO NPs showed toxic and apoptotic impacts on breast cancer cells. It was found that treatment of ZnO NPs with dosages of 5, 10, and 20 μM/mL for 48 h led to a significant decrease in cancer cell viability and also caused a concentration-dependent elevation in mRNA expression and protein level of p53 and Bax, while reducing the expression of Bcl-2 and ER-α [75]. The molecular mechanism of ZnO NPs on cancer cells has been studied. It has been shown that the treatment with ZnO NPs showed anticancer activity on bladder cancer T24 cells. In addition, low concentrations of ZnO NPs resulted in cell cycle arrest at the S phase, facilitated cellular late apoptosis, and repressed cell invasion and migration after 48-h exposure. These anticancer effects could be attributed to increased RUNX3 levels resulting from reduced H3K27me3 occupancy on the RUNX3 promoter, as well as decreased contents of histone methyltransferase EZH2 and the trimethylation of histone H3K27 [76]. In addition, ZnO NPs could penetrate into the cytoplasm and nucleus of T24 cells, which may result in the alteration of histone methylation [76]. In our study, we observed structural changes in the cancer cell nuclei after the treatment with NPs as shown by DAPI staining. The safety of ZnO NPs is critical for their therapeutic applications, as ZnO NPs are frequently used in biomedical research, including in cellular imaging and drug delivery [77]. In one study, it was found that treatment of ZnO NPs induced less cytotoxicity against normal lung-derived cells and did not elicit observable adverse effects after intravenous administration [77]. In our study, we also found that NPs induced less cytotoxic effects on the normal HEK-293 cells when compared to cancer cells.

## 5. Conclusions

In the current study, we reported the influence of Ce and Yb doping on the biological activities of ZnO nanoparticles. The structure, morphology, antifungal, and antibacterial actions against *C. albicans* and *E. coli* as well as the anticancer activities of these nanocomposites were studied for the first time. The different specimens were successfully fabricated by the sol-gel autocombustion process. The formation of nanoparticles having a hexagonal wurtzite phase was confirmed by XRD analysis. SEM and TEM observations supported XRD results. From this analysis, the average particle size decreased from 56.3 nm for pure samples (x = 0.00) to 41.4, 32.03, and 28.82 nm for co-doped samples with x = 0.01, x = 0.03, and x = 0.05 of Yb and Ce elements, respectively. The anticancer analysis showed a considerable reduction in the cell viability after treating for 48 h with Yb and Ce co-doped ZnO NPs. For pristine ZnO (x = 0.00), the cell viability was 68%, while for Yb and Ce co-doped ZnO samples, the cell viability was 58.70%, 80.94% and 64.91%, for x = 0.01, x = 0.03, and x = 0.05, respectively. We found that samples doped with x = 0.01 and x = 0.05 of Yb and Ce elements displayed better inhibitory actions on the cancer cells than the pure ZnO (x = 0.00). The IC_50_ of Yb and Ce ZnO nanoparticles was 3.50 µg/mL to 21.50 µg/mL for cancerous cells. The impact of Ce and Yb co-doping nanoparticles on normal HEK-293 cells was examined, and the cell viability was 86%, which is higher than that obtained for colon HCT-116 cells cancer (68%). In addition, DAPI staining analyses showed that the treatment of Ce and Yb co-doped nanoparticles caused apoptotic cell death in the HCT-116 cells. The prepared nanoparticles were also tested for their antimicrobial activities. Our results showed that Ce and Yb co-doped ZnO nanoparticles possess good anticancer and antimicrobial abilities. Further research is required to investigate the molecular mechanisms of these nanoparticles causing microbial and cancer cell death.

## Figures and Tables

**Table 1:** Structural parameters deduced from the XRD analysis.

Samples	x = 0.00	x = 0.01	x = 0.03	x = 0.05
$a\;\left(Å\right)$	3.2491	3.2501	3.2515	3.2533
$c\;\left(Å\right)$	5.2069	5.2048	5.2053	5.2080
**V = 0.866a^2^c** (${Å}^{3}$)	47.601	47.611	47.657	47.735
**D (nm)**	23.76	21.33	19.34	18.09

**Table 2:** MIC/MFC/MBC of *C. albicans* and *E. coli* upon treatment with Yb and Ce co-doped ZnO NPs (x = 0.00, x = 0.01, x = 0.03, and x = 0.05).

Samples		x = 0.00	x = 0.01	x = 0.03	x = 0.05
***C. albicans***	MIC (mg/mL)	1	0.25	0.25	0.25
	MFC (mg/mL)	>4	>4	>4	>4
***E. coli***	MIC (mg/mL)	2	1	1	0.5
	MBC (mg/mL)	>4	>4	4	4

## Data Availability

Not applicable.
